# IgE Epitope Mapping of Cross-Reactive Pollen, Fruit, and Vegetable PR-10 Allergens

**DOI:** 10.51894/001c.143508

**Published:** 2025-09-30

**Authors:** Andrea O’Malley, Brooke T. Borders, Jonas Lidholm, Ronald van Ree, Scott A. Smith, Maksymilian Chruszcz

**Affiliations:** 1 College of Osteopathic Medicine Michigan State University; 2 College of Osteopathic Medicine Michigan State University

## 04

### INTRODUCTION

PR-10 (pathogenesis-related group 10) allergens, also known as Bet v 1-like allergens, are implicated in pollen-food allergy syndrome (PFAS), which is caused by immunoglobulin E (IgE)-mediated cross-reactivity between pollen and fruit or vegetable allergens. The major birch allergen Bet v 1 is estimated to be responsible for IgE-mediated allergies in 100 million people worldwide. Information on human IgE epitopes on PR-10 allergens is very limited.

### OBJECTIVES

The main objective of this study is to map IgE-binding epitopes on cross-reactive PR-10 allergens. This will improve the understanding of PFAS as well as the pattern of occurrence of PR-10 related pollen and food allergies. Furthermore, studies of IgE-PR-10 interactions may lead to improved allergy treatment, such as with the use of hypoallergenic PR-10 proteins.

### METHODS

Human IgE (hIgE) monoclonal antibody (mAb) 3B6 was isolated from hybridomas produced by B cells from a subject with allergic rhinitis to pollen. Selected PR-10s were produced in E. coli and used for evaluation of hIgE mAb 3B6 specificity with ELISA EC50 assays and for structural studies with 3B6 Fab. Residues involved in the IgE-binding epitope observed in structures were selected for mutation. The mutants/hypoallergens were generated and tested for reduced binding with hIgE mAb 3B6 with ELISA EC50 assays.

### RESULTS

hIgE mAb 3B6 was found to bind recombinant oak, carrot, strawberry, and peanut PR-10s with high affinity. Structures were determined of 3B6 Fab in complex with Fra a 1.0102 (strawberry), Dau c 1.0103 (carrot), or Api g 1.0101 (celery). The IgE binding epitope was evaluated, and allergen residues forming strong interactions with the Fab were selected for mutation. Mutants of Fra a 1.0102 were produced in E. coli and tested for correct protein folding, and binding assays demonstrated their reduced to eliminated binding of hIgE mAb 3B6.

### CONCLUSIONS

3B6 Fab in complex with PR-10 allergens demonstrates a highly conserved IgE binding epitope on multiple proteins, structurally revealing the first cross-reactive hIgE epitope. Structural characterization of IgE Fabs in complex with PR-10 allergens will guide the development of hypoallergens and subsequent treatment of allergic conditions caused by sensitization to PR-10 allergens.

